# Identification of *Bacillus* Strains for Biological Control of Catfish Pathogens

**DOI:** 10.1371/journal.pone.0045793

**Published:** 2012-09-21

**Authors:** Chao Ran, Abel Carrias, Malachi A. Williams, Nancy Capps, Bui C. T. Dan, Joseph C. Newton, Joseph W. Kloepper, Ei L. Ooi, Craig L. Browdy, Jeffery S. Terhune, Mark R. Liles

**Affiliations:** 1 Department of Biological Sciences, Auburn University, Auburn, Alabama, United States of America; 2 Novus International Inc., Novus Aqua Research Center, Ho Chi Minh City, Vietnam; 3 Department of Pathobiology, Auburn University, Auburn, Alabama, United States of America; 4 Department of Entomology & Plant Pathology, Auburn University, Auburn, Alabama, United States of America; 5 Novus International Inc., Charleston, South Carolina, United States of America; 6 Department of Fisheries and Allied Aquacultures, Auburn University, Auburn, Alabama, United States of America; Universidad Nacional Autonoma de Mexico, Instituto de Biotecnologia, Mexico

## Abstract

*Bacillus* strains isolated from soil or channel catfish intestine were screened for their antagonism against *Edwardsiella ictaluri* and *Aeromonas hydrophila,* the causative agents of enteric septicemia of catfish (ESC) and motile aeromonad septicaemia (MAS), respectively. Twenty one strains were selected and their antagonistic activity against other aquatic pathogens was also tested. Each of the top 21 strains expressed antagonistic activity against multiple aquatic bacterial pathogens including *Edwardsiella tarda*, *Streptococcus iniae*, *Yersinia ruckeri*, *Flavobacterium columnare*, and/or the oomycete *Saprolegnia ferax.* Survival of the 21 *Bacillus* strains in the intestine of catfish was determined as *Bacillus* CFU/g of intestinal tissue of catfish after feeding *Bacillus* spore-supplemented feed for seven days followed by normal feed for three days. Five *Bacillus* strains that showed good antimicrobial activity and intestinal survival were incorporated into feed in spore form at a dose of 8×10^7^ CFU/g and fed to channel catfish for 14 days before they were challenged by *E. ictaluri* in replicate. Two *Bacillus subtilis* strains conferred significant benefit in reducing catfish mortality (P<0.05). A similar challenge experiment conducted in Vietnam with four of the five *Bacillus* strains also showed protective effects against *E. ictaluri* in striped catfish. Safety of the four strains exhibiting the strongest biological control *in vivo* was also investigated in terms of whether the strains contain plasmids or express resistance to clinically important antibiotics. The *Bacillus* strains identified from this study have good potential to mediate disease control as probiotic feed additives for catfish aquaculture.

## Introduction

Aquaculture farming of the channel catfish, *Ictalurus punctatus*, has been one of the most successful animal production industries in North America in the past 30 years and currently represents the largest aquaculture industry in the United States. Over 90% of all catfish produced in the U.S. are raised in Alabama, Arkansas, Louisiana, and Mississippi and are primarily grown in earthen ponds ranging in size from 2 to 10 ha [1], [2].

Feed inputs associated with high-density fish culture stimulate the proliferation of opportunistic bacteria [3]. The combination of high-density fish culture with rapidly changing water temperature and chemical composition of aquaculture ponds places stress on fish, thereby resulting in favorable conditions for the onset and spread of disease. Enteric Septicemia of Catfish (ESC), caused by the Gram negative bacterium *Edwardsiella ictaluri* [4], is the most important endemic infectious disease in the channel catfish aquaculture industry [5]. Losses resulting from ESC were reported in over 78% of all operations with outbreaks being reported in 42% of catfish production ponds, with an economic loss between $20 and $30 million yearly [1], [2], [6].

Another important pathogen in channel catfish is *Aeromonas hydrophila*, which is the primary causative agent of motile aeromonad septicaemia (MAS) [7] and can infect multiple fish species including tilapia, catfish, goldfish, common carp, and eel [8]. In 2009 and 2010, *A. hydrophila* was identified as the etiologic agent of a disease epidemic in farmed channel catfish, resulting in higher mortality rates than typical for MAS with over five million pounds of catfish lost in the Alabama commercial catfish industry. The *A. hydrophila* strains (e.g., strain AL09-119) isolated from diseased fish during this epidemic are highly virulent in aquaria disease challenge trials compared to *A. hydrophila* reference strains [8].

*Pangasianodon hypophthalmus* Sauvage, commonly known as the striped catfish, is the native catfish in the Mekong Delta of Vietnam. The farming sector of *P. hypophthalmus* has recorded the highest growth rate in volume compared to any other aquaculture commodity globally over the last decade [9], [10]. The sector accounted for 687,000 and 1,094,879 t production in 2007 and 2008, respectively, the latter amounting to 34% of the total aquaculture production in Vietnam, the fifth-ranked nation in global aquaculture production [11]. Furthermore, over 90% of the farmed catfish is processed and exported to more than 100 countries globally [10]. Bacillary necrosis of *Pangasius* spp. (BNP), also caused by *E. ictaluri*, is an economically significant disease for the striped catfish aquaculture industry in the Mekong Delta, which can cause 50–90% mortality and occurs in 98% of farms [9].

Chemotherapy by oral administration of antibiotics in fish feeds is the most common treatment for bacterial diseases. However, the indiscriminant application of antibiotics may result in many problems including the spread of drug-resistant pathogens, environmental hazards and food safety problems. This has fostered an increased interest in alternatives to antibiotics. Probiotics, which have various health-promoting properties and minor adverse side effects, are gaining an increasing scientific and commercial interest in aquaculture practice. The beneficial effects of probiotics involve improvement of feed utilization, modulation of intestinal microflora, enhancement of immune responses and antagonism to pathogens. The most commonly used probiotics in aquaculture are lactic acid bacteria and *Bacillus* spp. [12]. *Bacillus* species have advantages as probiotics in that their spore-forming ability allows greater viability after pelleting and high survival rates after exposure to gastric acid [13], [14], [15]. *Bacillus* spp. has been reported to have various beneficial attributes when applied to fish [16], [17], [18], [19], [20], [21], [22].

Few studies have been conducted to investigate probiotic bacteria for mitigating infectious diseases in channel catfish, and no studies have been reported using direct administration in feed. Queiroz and Boyd [23] applied a commercial probiotic product, Biostart, which contained a few species of *Bacillus* spp., to channel catfish pond water and demonstrated that survival and net production of fish treated with *Bacillus spp.* were significantly greater than the control. However, the bacteria used in this previous study were not isolated specifically for use in channel catfish nor were their antimicrobial activities against important pathogens of channel catfish characterized.

In this research an extensive collection of *Bacillus* strains (n = 160) isolated from soil and from the intestine of channel catfish (n = 17) was tested for *in vitro* antimicrobial activity against *E. ictaluri, A. hydrophila*, and other bacterial and fungal pathogens of channel catfish. *Bacillus* strains that showed effective antibiosis were evaluated for their respective survival in the intestine of channel catfish. The biological control activity of the best performing *Bacillus* strains when amended onto feed was investigated using channel and striped catfish disease challenge studies in an aquarium system. The safety of selected *Bacillus* strains was also assessed in terms of the presence of plasmids and resistance to antibiotics.

## Materials and Methods

### Ethics Statement

All experiments conducted with vertebrate animals (catfish) were approved by the Institutional Animal Care and Use Committee (IACUC) review boards at Auburn University or Novus International Inc. in accordance with the respective animal welfare guidelines specified in the United States or Vietnam.

### Bacterial Strains

*E. ictaluri* strain S97-773 was used for the primary screening for *Bacillus* antibiosis and for ESC challenge experiments since this strain is highly pathogenic for channel catfish, has previously been used in challenge studies and was obtained from the Southeastern Cooperative Fish Disease Laboratory (SCFDL), Auburn University. *E. ictaluri* strain R-4383, *E. ictaluri* strain Alg-08-200, *E. tarda* AL09-82, *S. iniae* USDA 2009, *Y. ruckeri* ATCC 29473, and *F. columnare* AL09-10 were from the collection of pathogenic isolates at the SCFDL, and *S. ferax* was obtained from Carolina Biological (catalog # 156271, Burlington, NC). *E. ictaluri* NLF33 were isolated from diseased striped catfish in Vietnam. *A. hydrophila* ML09-119 was isolated from a diseased channel catfish with MAS in 2009. The collection of soil-derived *Bacillus* strains (n = 160) was provided by the laboratory of Dr. Joseph Kloepper (Department of Entomology and Plant Pathology, Auburn University). *Bacillus subtilis* 1E17 was obtained from the *Bacillus* Genetic Stock Center (http://www.bgsc.org/).

### Isolation of *Bacillus* spp. Strains from the Intestine of Channel Catfish and Evaluation of Antimicrobial Activity

Healthy catfish (7–10 cm) were killed by administration of an overdose of MS-222 (tricaine mesylate, FDA approved for anesthesia in fin fish), and the digestive tracts were removed in their entirety. Approximately 1.0 g of the intestinal tissue with gut content was homogenized in 9.0 ml of sterile saline (0.9% w/v) using a sterile mortar and pestle. Ten-fold serial dilutions were prepared to 10^−6^ in fresh 1x phosphate-buffered saline (PBS), and 0.1 ml was spread over the surface of triplicate plates of tryptone soy agar (TSA) with incubation at 28°C for 48 h [24]. *Bacillus*-like colonies were picked at random, purified by streaking for isolated colonies on fresh media, and examined for inhibition against the growth of *E. ictaluri* using the double-layer soft agar method [25]. For the soft agar overlay, the bacterial isolates were grown in 5 ml of tryptone soy broth (TSB) for 24 h at 30°C. A volume of 5 µl was then spotted onto triplicate plates of TSA and incubated for a further 24 h. Soft agar (0.7% w/v agar) prepared with TSB was melted, cooled and seeded with an inoculum of log-phase *E. ictaluri* strain S97-773 to achieve slight turbidity (i.e., ∼10^7^ cells/ml). The bacterial cell suspension in soft agar was immediately poured over the TSA plates and incubated for 24 h at 30°C whereupon the presence of zones of clearing in the growth of of *E. ictaluri* were recorded (in mm) as evidence of growth inhibition. Cultures that were regarded as inhibitory to *E. ictaluri* were characterized by Gram staining and 16S rRNA gene sequencing using the ‘universal bacteria’ primer set 27F and 1492R [26]. A consensus 16S rRNA sequence was produced using ChromasPro (Technelysium Pty Ltd., Queensland, Australia), and each sequence was compared to the GenBank non-redundant nucleotide database by BLASTn. *Bacillus* spp. strains were cryopreserved at −80°C. The collection of soil-derived *Bacillus* strains (n = 160) was tested for antimicrobial activity against *E. ictaluri* using the same method.

Fifty *Bacillus* strains with antagonistic activity against *E. ictaluri* S97-773 were tested for their inhibitory activity against other *E. ictaluri* strains (*E. ictaluri* R-483, *E. ictaluri* Alg-08-200). *Bacillus* strains that showed antimicrobial activity against all three *E. ictaluri* strains were evaluated further for their activity to inhibit the growth of *A. hydrophila* strain AL09-119. Twenty-one *Bacillus* strains that showed significant antimicrobial activity against both *E. ictaluri* and *A. hydrophila* were tested for their activity against several other channel catfish pathogens including *E. tarda*, *S. iniae*, *Y. ruckeri*, *S. ferax* with the soft agar overlay method described above.

The antimicrobial activity against *F. columnare* was tested by an agar well diffusion method. For the well diffusion assay, the *Bacillus* strains were grown in 5 ml of TSB for 48 h at 30°C. After centrifugation at 3,600×g for 10 min, the culture supernatant was filtered through a 0.2 µm filter. Then 200 µl of the filter-sterilized supernatant was added to a round well (approx. 10 mm in diameter) made in a *F. columnare* growth medium (FCGM) agar plate [27]. After the supernatant was absorbed into the agar medium, a log-phase *F. columnare* culture grown in FCGM broth was spread thoroughly over the plate using a sterile cotton swab. The plates were incubated for 48 h at 30°C. The zones of clearing in the growth of the lawn of *F. columnare* were measured by the same method as in double-layer soft agar protocol.

*Bacillus* strains AB01, AP79, AP143, AP193, and AP254 were sent to Vietnam and their *in vitro* antimicrobial activity was tested against *E. ictaluri* NLF33, the causative agent of BNP in striped catfish. A broth culture of *E. ictaluri* was adjusted to 10^6^ CFU/mL and evenly swabbed onto TSA plates. Three wells were punched from the agar plate and 50 µL of a 10^8^ CFU/mL of a *Bacillus* cell-free supernatant (48 h culture in TSB) was added into each well. Zones of inhibition were measured after 24 hours incubation at 30°C.

### Preparation of *Bacillus* Spores and Spore-amended Feed

*Bacillus* spores were prepared by the method described by Kenny and Couch [28] with some modifications. *Bacillus* strains were grown in TSB at 30°C overnight. The cell suspension was spread onto spore preparation agar (peptone 3.3 g/l, beef extract powder 1.0 g/l, NaCl 5.0 g/l, K_2_HPO_4_ 2.0 g/l, KCl 1.0 g/l, MgSO_4_. 7H_2_O 0.25 g/l, MnSO_4_ 0.01 g/l, lactose 5 g/l, agar 15 g/l) using a sterile cotton swab and incubated at 28°C for 5 to 7 days. To collect the spores, 5 ml of sterile distilled water was added to the plate and the spores were suspended in water using an inoculation loop. The spore suspension was then incubated at 85°C for 15 min to kill the vegetative cells. The concentration of the spore suspension was determined by 10-fold serial dilution in 1 x PBS and spreading onto TSA. The final concentration of the spore suspension was manipulated with sterile water to 1.25×10^10^ CFU/ml for the intestinal survival assay and 10^9^ CFU/ml for the challenge study. To prepare spore-amended feed, 80 ml of the spore suspension was sprayed onto 1000 g commercially available slow-sinking pelleted fish feed (2 mm, 40% protein, Zeigler, Gardners, PA) using a bleach- and ethanol-sterilized pump sprayer to achieve approximately 8% (v/w) spore suspension application. The feed was then mixed thoroughly with 30 ml fish oil. The control feed was ammended solely with fish oil.

### Inoculation and Quantification of *Bacillus* spp. in the Intestine of Channel Catfish

Fingerling channel catfish (7–10 cm) were distributed into twenty-three 60 L tanks each containing 15 L water and three fish. Fish were starved for one week prior to the experiment. Catfish feed was amended in separate batches with the 21 *Bacillus* strains that showed good antimicrobial activity against both *E. ictaluri* and *A. hydrophila* using the spore application method described above. Each unique *Bacillus* strain-amended feed (∼10^9^ CFU/g feed) was given to one aquarium tank. The fish were fed once daily with spore-amended feed or control feed for one week, and thereafter all fish received the control feed for three days. One tank was used as the control and received untreated fish feed for the duration of the experiment. Daily feeding rate was 3% of total body weight.

At the end of the experiment, all of the fish were killed by administration of an overdose of MS-222. The intestine was removed, weighed, and then homogenized in sterile saline (0.9% w/v). The final volume of the homogenized intestine sample was adjusted to 2 ml by sterile saline. Homogenized samples were then serially diluted in sterile saline and spread on TSA and incubated at 28°C for 48 h. Three representative colonies with the same morphology as the applied *Bacillus* strain were randomly picked from the plate, purified on new plates and identified by 16S rRNA gene sequencing as described previously and compared with the known 16S rRNA gene sequence from each respective *Bacillus* strain. For the control and treatment groups, only the unique colony morphology corresponding to that of the amended *Bacillus* strains was recorded. Cultured counts for each *Bacillus* strain recovered from the intestine were determined as CFU/g of intestine sample.

### Aquarium Challenge Studies

In the first challenge, five *Bacillus* strains (AB01, AP143, AP193, AP254, and AP79) were selected for evaluation of their biological control of ESC in an aquarium challenge. Five *Bacillus* treatments and one control, each with four replicate aquaria, were included. Each replicate aquarium was stocked with 25 fingerling channel catfish weighing about 13 g. Fish were acclimated to commercial dry feed for one week. Fish from each treatment group were then fed with an experimental diet supplemented with spores of one *Bacillus* strain (8×10^7^ CFU/g) at a daily feeding rate of 2.5% fw/bw (feed weight/body weight) for two weeks. Fish in the control group received normal feed only.

Fish were challenged by immersion for 45 minutes in 10 L of water containing 4.5×10^6^ CFU/ml *E. ictaluri* S97-773. All fish from the same group were immersed in a single container. The challenge condition for the control group was the same as other treatments except that Brain Heart Infusion (BHI) medium was added instead of *E. ictaluri* culture. Mortalities were monitored over a 21-day period, and dead fish were dissected and the presence of *E. ictaluri* confirmed by microbiological examination of kidney and liver swabs on TSA. The final mortality was calculated when there was no more mortality for five consecutive days after challenge. The identity of the recovered *E. ictaluri* was confirmed by biochemical analyses that included indole and hydrogen sulfide production, catalase and oxidase activities, hydrolysis of esculin and gelatin, nitrate reduction, and acid formation from carbohydrates.

Fish were reared in a recirculating system during the acclimation period. Upon initiation of *Bacillus* feeding and during the challenge phase, a static system was incorporated with a 20–30 min daily water exchange that resulted in turnover of approximately half of the aquarium water. Sponge biofilters and daily removal of uneaten/waste materials were incorporated to improve water quality. Water temperature was kept at 26±2°C. During the static phase, the central room heating system in conjunction with submersible aquarium water heaters was used to control the required water temperature, and a water heater system was used to control the temperature of the incoming water during water exchange.

In a second challenge trial using channel catfish, flow-through conditions were used to reduce catfish mortality. In this challenge experiment, four *Bacillus* treatments (AP79, AP143, AP193, AB01) and one control each with four replicate aquaria were included. Each aquarium was stocked with 20 fingerling channel catfish (∼12 g). A lower dose of *E. ictaluri* S97-773 (8×10^5^ CFU/ml) was used to challenge fish and starting immediately after challenge the aquaria were flushed for 5–8 hours a day. All other conditions in this challenge were the same as described above. Mortalities were monitored over a 21-day period after challenge, and presence of *E. ictaluri* in the dead fish was confirmed as previously described.

A third challenge trial was conducted to evaluate the protective effect of four *Bacillus* strains (AP79, AP193, AP254 and AB01) against *E. ictaluri* for striped catfish. Four *Bacillus* treatments and one control each with four replicate tanks were included in this study. Each tank was stocked with 18 striped catfish (∼14 g). Striped catfish were administered feed amended with *Bacillus* spores (∼10^7^ CFU/g feed) or control feed for two weeks and the fish were transferred to 80 L tanks for a bath challenge with *E. ictaluri* NLF33. Fish were immersed for 30 min in static, aerated aquaria at a dose of ∼10^6^ CFU/mL to target about 70% mortality in the control group. The control and test diets were offered throughout the trial. The recording of mortality and confirmation of *E. ictaluri* in dead fish were conducted as above.

### Plasmid Analysis

Plasmid DNA was extracted from *Bacillus* strains AP79, AP143, AP193, and AB01 by the alkaline lysis method [29]. *Bacillus subtilis* 1E17 containing plasmid pC194 was used as a positive control. The extracted DNA was analyzed by a Chef-DR II pulsed field electrophoresis system (Bio-Rad, Hercules, CA). Pulse time ranged from 1 to 15 seconds for 15 h at 6 V/cm. The gel was stained with ethidium bromide and visualized using an AlphaImager HP gel documentation system (ProteinSimple, Santa Clara, CA).

### Antibiotic Resistance Analysis

The susceptibility of *Bacillus* strains AP79, AP143, AP193, and AB01 to carbenicillin, ampicillin, spectinomycin, oxacillin, vancomycin, cephalothin, novobiocin, sulfadiazine, amikacin, erythromycin, neomycin, penicillin, chloramphenicol, sulfamethoxazole, norfloxacin, gentamicin and ciprofloxacin was determined by disc diffusion test following procedures outlined by National Committee for Clinical Laboratory Standards [30]. A log-phase culture of each strain was diluted to a concentration of approximately 1×10^8^ to 2×10^8^ CFU/ml (McFarland standard 0.5). The inoculum was then seeded onto a Mueller-Hinton agar plate using a cotton swab. Antibiotic-impregnated discs (BD Biosciences) were placed on seeded plates, and the diameter of the zone of growth inhibition was measured after 18 h of incubation at 37°C. The experiments were repeated three times and the average diameter of inhibition zones was calculated.

### Statistics

A completely randomized design was used in this research. Data were presented as mean ± standard error (SE). Challenge data were subjected to analysis of variance in SAS 9.2. Differences between means were tested by Tukey’s range test and were considered significant when probability (P) values < 0.05 were obtained.

## Results

### Characterization of *Bacillus* Isolates

Each of the *Bacillus* strains isolated from soil or catfish intestine that exhibited inhibitory activity against both *E. ictaluri* and *A. hydrophila* was capable of endospore formation. Each pure *Bacillus* culture was ribotyped, indicating that most of the *Bacillus* strains were within the *B. subtilis* group (inclusive of *B. amyloliquefaciens*) (Table 1). The 16S rRNA gene sequences for the 21 strains were submitted to GenBank (Accession Numbers JX094283 to JX094303). Two strains of *B. pumilus* (AP18 and AP280) and one strain of *B. methylotrophicus* (AP191) were also within the collection. Strain AP76 was identified as *Bacillus cereus* and thus eliminated for further evaluation due to the potential for foodborne illness. All of the *Bacillus* strains that were subsequently determined to have *in vivo* biological control activity were within the *B. subtilis* group. This phylogenetic affiliation was also confirmed by comparison of genome sequences to the GenBank nr/nt database for strains AB01, AP79, AP143, and AP193, indicating >80% average nucleotide identity to a previously sequenced genome within the *B. subtilis* group (data not shown).

**Table 1:** Antimicrobial activity of 22 *Bacillus* strains against multiple aquatic pathogens.

Phylogeny	Strain	*Aeromonas hydrophila*	*Edwardsiella ictaluri*	*Edwardsiella tarda*	*Flavobacterium columnare*	*Saprolegnia ferax*	*Streptococcus iniae*	*Yersinia ruckeri*
*B. subtilis* group	AB01	**+**	**+**	**+**	**+++**	**+**	**++**	**++**
*B. pumilus*	AP18	**+**	**+**	**-**	**−**	**+**	**+**	**−**
*B. subtilis* group	AP71	**+**	**++**	**+**	**−**	**−**	**++**	**+**
*B. cereus*	AP76	**+**	**++**	**++**	**−**	**+**	**++**	**++**
*B. subtilis* group	AP77	**+**	**+++**	**++**	**−**	**+**	**+**	**++**
*B. subtilis* group	AP79	**++**	**++**	**+**	**−**	**−**	**++**	**++**
*B. subtilis* group	AP102	**+**	**++**	**++**	**++**	**+**	**−**	**++**
*B. subtilis* group, *B. amyloliquefaciens* ‡	AP143	**++**	**++**	**++**	**−**	**+**	**+**	**++**
*B. subtilis* group	AP183	**+**	**++**	**++**	**−**	**−**	**++**	**+**
*B. subtilis* group	AP189	**++**	**+++**	**++**	**−**	**−**	**++**	**+**
*Bacillus methylotrophicus*	AP191	**++**	**+++**	**+**	**+**	**+**	**++**	**−**
*B. subtilis* group, *B. amyloliquefaciens* ‡	AP193	**++**	**++**	**++**	**++**	**+**	**+**	**−**
*B. subtilis* group	AP215	**+**	**+++**	**+**	**−**	**+**	**+**	**++**
*B. subtilis* group	AP218	**+**	**++**	**+**	**−**	**+**	**+**	**+**
*B. subtilis* group	AP219	**++**	**++**	**+**	**+**	**+**	**++**	**+**
*B. subtilis*	AP254	**+**	**++**	**+**	**++**	**−**	**−**	**−**
*B. pumilus*	AP280	**+**	**++**	**+**	**−**	**+**	**+**	**+**
*B. subtilis* group	AP295	**+**	**++**	**+**	**−**	**+**	**+**	**++**
*B. subtilis* group	AP301	**+**	**++**	**+**	**+++**	**+**	**++**	**+**
*B. subtilis* group	AP303	**++**	**++**	**++**	**−**	**+**	**+**	**++**
*B. subtilis* group	AP305	**++**	**++**	**++**	**−**	**−**	**+**	**++**

‡ Draft genome sequences are available for these *Bacillus* strains, so the phylogenetic affiliation is inferred from a comparison of these *Bacillus* strain genome sequences with previously sequenced *Bacillus* genomes.

Note that (+) indicates a zone of inhibition up to 5 mm, (++) indicates a zone of inhibition from 5 mm to 1 cm, (+++) indicates a zone of inhibition greater than 1 cm, and (−) indicates no observable zone of inhibition.

### Antimicrobial Activity of *Bacillus* Strains

The *B. subtilis* strain AB01 isolated from the catfish intestine showed significant antimicrobial activity against *E. ictaluri*. From the collection of soil-derived *Bacillus* strains, 49 strains showed significant antagonism against *E. ictaluri.* All of the 50 *Bacillus* strains also showed inhibitory activity against *E. ictaluri* R-4383 and *E. ictaluri* Alg-08-200. A total of 21 *Bacilllus* strains showed potent antibiotic activity against both *E. ictaluri* and *A. hydrophila* (Table 1). The 21 *Bacillus* strains selected were tested for their activity against multiple pathogens in aquaculture. All of the strains were antagonistic against multiple catfish pathogens, including Gram-negative and -positive bacteria, and the oomycete *Saprolegnia*. *Bacillus* strains AB01, AP219, and AP301 showed antimicrobial activity against all of the tested pathogens (Table 1). Also, all five of the *Bacillus* strains tested for biological control of BNP (AP79, AP143, AP193, AP254, AB01) showed significant antagonistic activity against *E. ictaluri* NLF33 (data not shown).

### Survival and Persistence of *Bacillus* Strains in the Intestine of Channel Catfish

**Figure 1:**
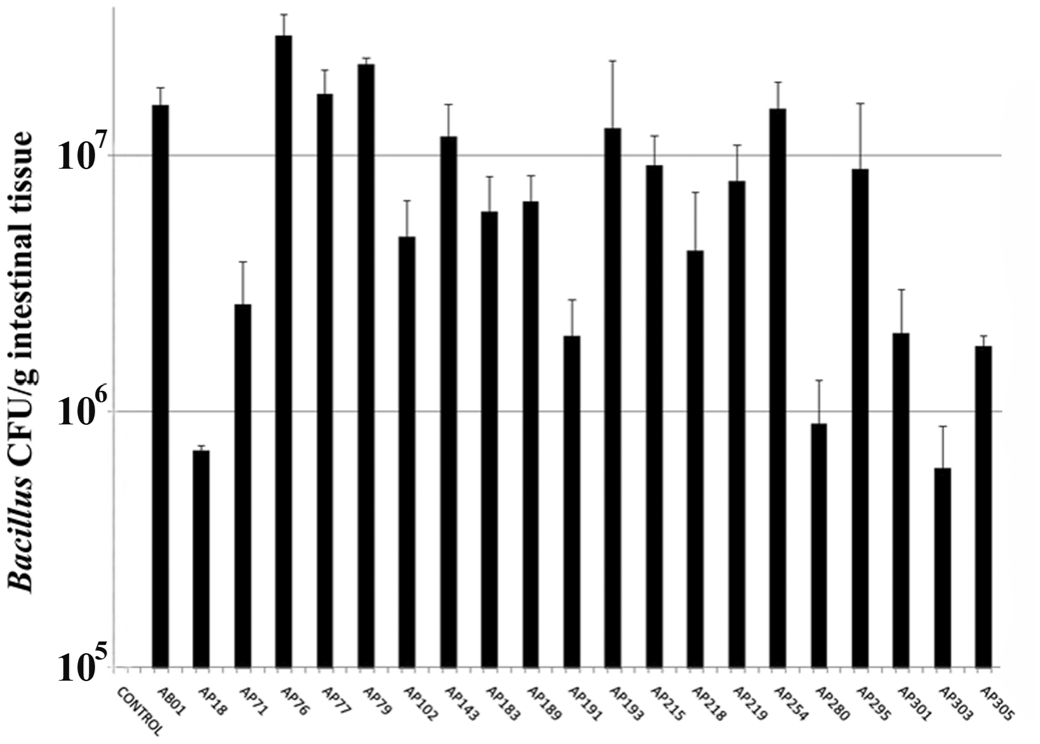
*Bacillus* strain CFUs/g of catfish intestine, after feeding with *Bacillus*-amended or non-amended feed (n = 3 animals per *Bacillus* strain).

*Bacillus* spores administered to channel catfish via feed for one week followed by three days of control feed were recovered from the catfish intestine. Over 10^7^ CFU/g of introduced *Bacillus* was observed in the gut for strains AB01, AP76, AP77, AP79, AP143, and AP254 (Fig. 1). For strains AP18, AP280, and AP303, the counts of recovered bacteria were relatively low, and they were eliminated from further investigation. None of the 21 *Bacillus* strains were recovered from the control group. In all cases the 16S rRNA gene sequence determined from representative colonies matched the 16S rRNA gene sequence from the respective *Bacillus* strain that was added to catfish feed (data not shown).

### Challenge Study

**Figure 2:**
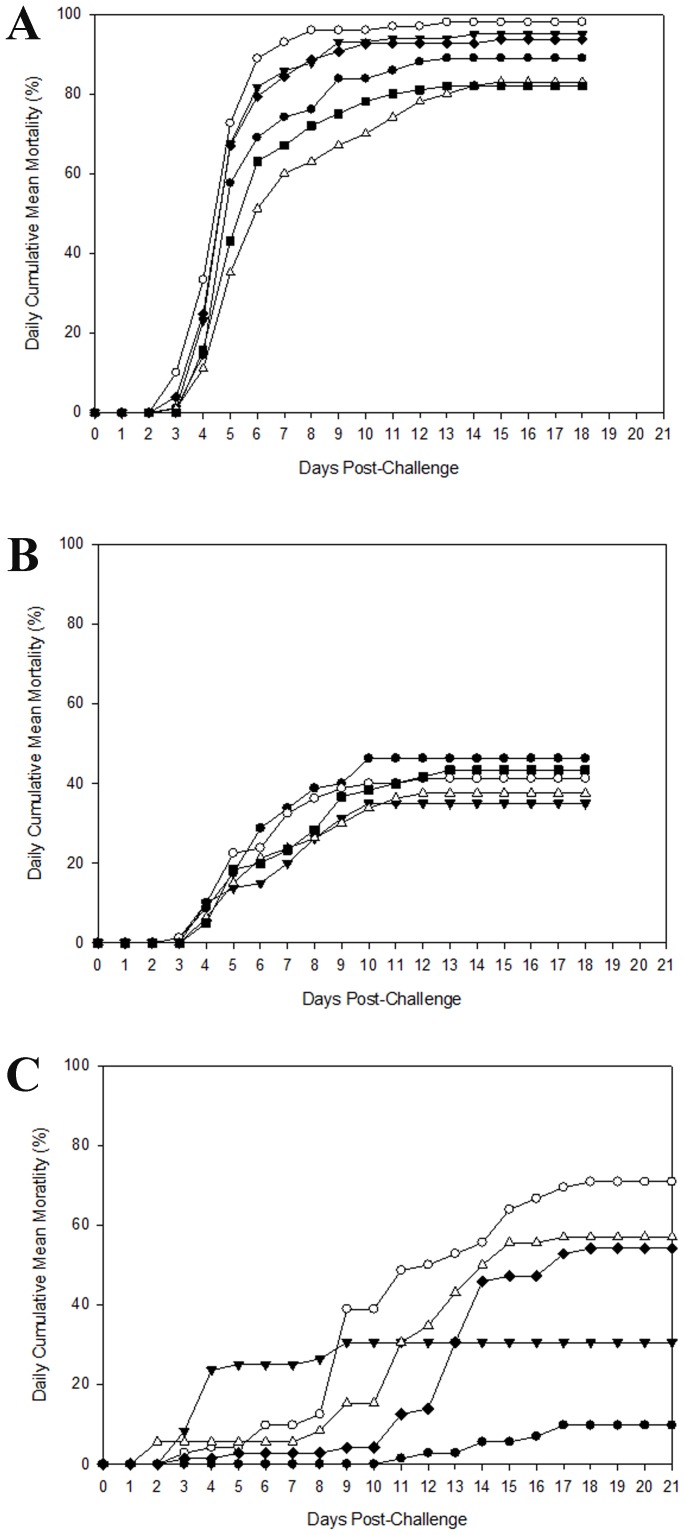
Daily mean cumulative mortality of (A) channel catfish in static system with 20–30 min daily water exchange and (B) channel catfish with 5–7 h flow through water daily, or (C) striped catfish in static system with 20–30 min daily water exchange, fed with and without addition of *Bacillus* strains and challenged with *E. ictaluri*. All values are means of four replicates per treatment. Treatments: (open circle) Control, (closed circle) AP79, (closed triangle) AP193, (open triangle) AB01, (closed square) AP143, and (closed diamond) AP254.

In the first immersion challenge, the mean mortality of control group was 98.0%, a very high level of mortality that was likely a consequence of the persistence of *E. ictaluri* within the aquaria water under static conditions. Treatment groups that were fed with feed amended with spores of *Bacillus* strains AP143 or AB01 showed significantly reduced mortality compared with the control (P<0.05), with 83.1% and 84.8% mortality, respectively. There was no significant difference in the mortality observed between the two strains. The treatment groups fed with *Bacillus* strains AP79, AP193, or AP254 (with mortality 89.0%, 95.0%, and 93.7%, respectively) did not show significant differences compared with the control (Fig. 2A, Table 2). In every challenge experiment a control group was included that was not challenged with *E. ictaluri*, and in every case this control group had no observed mortalities, demonstrating that mortalities were due to infection with *E. ictaluri*.

**Table 2:** Mortality (%) (± SE) of groups of fish that received feed amended with different *Bacillus* strains or control feed and were challenged with *E. ictaluri* (n = 4).

Treatment	Channel catfish challenge (Fig. 2A)	Channel catfish challenge (Fig. 2B)	Striped catfishchallenge (Fig. 2C)
Control	98.0±1.2^a^	41.3±5.9^a^	70.8±7.3^a^
AB01	84.8±2.0^bc^	37.5±9.5^a^	56.9±6.6^ab^
AP143	83.1±2.9^bc^	43.3±14.8^a^	Not determined
AP193	95.0±3.0^ab^	35.0±5.4^a^	30.6±23.7^ab^
AP254	93.7±2.8^ab^	Not determined	54.2±11.4^ab^
AP79	89.0±2.7^abc^	46.3±5.2^a^	9.7±6.6^b^

Means in the same column sharing a common superscript letter were not significantly different.

(P>0.05 ) as determined by Tukey’s test.

For the second immersion challenge, flow-through conditions were adopted post-challenge to reduce catfish mortality, and this was successful as only 41.3% mortality was observed in the control group. However, under the flow-through conditions of this challenge the mortality in the treatment groups ranged from 35.0% to 46.3% with no significant differences observed between any of the treatment groups and the control (Fig. 2B, Table 2).

In the striped catfish challenge experiment, the catfish were again challenged by immersion and then maintained under static conditions similar to those used in the first channel catfish challenge. Under these conditions the control group had 70.8% mortality which is consistent with the higher mortality observed under static conditions when re-infection can occur (Fig. 2C, Table 2). The treatment group fed with feed amended with spores of strain AP79 had the lowest (9.7%) cumulative mortality and this was significantly different from the control (P<0.05). Catfish fed with spores of strains AP193, AP254 or AB01 had 30.6%, 54.2% and 56.9% mortality, respectively.

### Plasmid and Antibiotic Resistance Study

An analysis of plasmid DNA extracted from *Bacillus* strains AP79, AP143, AP193, and AB01 was conducted by PFGE. We did not observe the presence of any plasmid within AP79, AP193 and AB01 but the positive control did show the presence of plasmid pC194 (data not shown). A plasmid was evident from strain AP143. High coverage contigs from the assembled genome sequence of AP143 (data not shown) were screened by BLASTn and one of the contigs showed high identity (99%) with plasmid pBSG3 from *Bacillus amyloliquefaciens* B3 [31]. BLASTx analysis of the predicted open reading frames from this plasmid did not show any similarity to genetic loci involved in antibiotic resistance or virulence. Evaluation of antibiotic susceptibility determined that all of these strains were susceptible to all of the tested antibiotics to varying degrees. They were all highly susceptible to carbenicillin, cephalothin, sulfamethoxazone and ciprofloxacin (>25 mm diameter inhibition zone). Ampicillin, penicillin, vancomycin, novobiocin, amikacin, erythromycin, neomycin, chloramphenicol, norfloxacin and gentamicin also inhibited their growth effectively (20–25 mm zones of inhibition), whereas spectinomycin, oxacillin, sulfadiazine showed moderate inhibition (15–20 mm inhibition zones). These strains showed very similar antibiograms, with the variation in the diameter of inhibition zones less than 10% of the average diameter for each of the antibiotics tested.

## Discussion

The results of this study indicate that specific strains within the *Bacillus subtilis* group showed promise for biological control of disease in catfish aquaculture. It provided another evidence of the efficacy of the *Bacillus* strains in boosting disease resistance of fish [16], [17], [19], [20]. This study is the first to select probiotic bacteria for control of ESC and other pathogens in catfish and to evaluate their biocontrol efficacy via feed administration.

Gatesoupe [32] concluded that probiotics for aquaculture should be antagonistic to pathogens, colonize intestines, and increase resistance of the host to pathogens. Ideally probiotic bacteria should be selected by considering all three criteria. However, it is difficult to evaluate potential probiotic bacterial strains for the second and third criterion on a large number of candidate bacteria. Therefore, *in vitro* antimicrobial activity was the primary criteria by which a large number of strains were evaluated, with candidate bacterial strains that did not show antagonistic activity eliminated from further study. The primary objective of this research project was to identify bacterial strains that can be applied for the control of *E. ictaluri, A. hydrophila*, as well as other bacterial and oomycete pathogens of catfish. Since the bacterial pathogens *E. ictaluri* and *A. hydrophila* are responsible for the majority of the mortality observed currently in catfish aquaculture, the ability of a *Bacillus* strain to inhibit the growth of these two pathogens was of paramount importance and only the strains capable of inhibiting both pathogens were selected for testing in aquarium disease challenges.

The ability of a probiotic bacterial strain to colonize and survive within or on its host is also an important criterion for strain selection. However, in many cases the probiotic bacteria may not permanently colonize the gastrointestinal tract but instead achieve a sustained transient state [24], [33]. Even transient bacteria may be efficient at mediating biological control of disease if the cells are introduced artificially via food either continuously or semi-continuously [32], [34]. High population levels of several *Bacillus* strains were recovered from catfish intestines three days post-feeding with *Bacillus*-spore amended feed. For *Bacillus* strains with high counts in the intestine, colonies with the same morphology as the applied *Bacillus* strain dominated the TSA plates, and the ribotype of the representative colonies confirmed their identity as the applied *Bacillus* strain. Considering that bacterial population levels in the intestine should decline after cessation of feeding with the spore-containing diets, the maximal level of *Bacillus* strain CFU/g of intestinal tissue reached during the feeding regime may be higher. The bacterial population levels here (10^6^–10^7^ CFU/g for most of the strains) are in general agreement with previous studies involving fish [24], [33], [35], [36]. These results demonstrate that some of the *Bacillus* strains evaluated in this study can persist within the catfish gastrointestinal tract for at least three days. However, at this point the degree of persistence and ability to colonize the intestinal mucosa are unknown for each strain. A more detailed experiment evaluating the colonization and/or persistence of specific *Bacillus* strains within the catfish intestine will be conducted to help understand the biocontrol mechanism(s) of *Bacillus* strains and guide the duration and timing of *Bacillus* feeding. Future studies will also examine the impact of each *Bacillus* strain on the intestinal microbiota and the health and growth of the fish in the absence of aquaculture pathogens.

Three aquarium disease challenges were conducted in this study, two of which evaluated biological control of ESC in channel catfish. In the first channel catfish disease challenge, a very high mortality (98.0%) due to ESC was observed in the control group. Ideally, an aquarium disease challenge would result in a mortality of 60%–70%, which more accurately simulates the natural development of ESC in an aquaculture pond. The high mortality was probably the consequence of the incorporation of a static system after the immersion challenge, wherein the *E. ictaluri* persisted in the aquaria for an extended period of time and bacterial cells shed in feces could potentially infect other fish within the same aquarium. Despite the higher mortality observed in this first challenge, two *Bacillus* strains (AP143, AB01) provided significant protection to channel catfish. However, the degree of mortality reduction for the *Bacillus* treatment groups compared with control was lower in the first challenge compared to a later challenge with striped catfish. This discrepancy might be due to the much higher mortality in the first challenge, which could have reduced the biological control capacity of the *Bacillus* strains. Presumably the degree of biological control would be greater in an ESC challenge if lower mortality (∼70%) was obtained in the control group. In addition, at the lower doses of *E. ictaluri* that catfish are typically exposed to in an aquaculture pond the degree of biocontrol provided by *Bacillus* strains would presumably be of an even greater magnitude.

One solution to reduce channel catfish mortality during an aquarium disease challenge is to use a flow-through system, but we discovered that the use of a flow-through system also resulted in the loss of *Bacillus* biological control activity. The lack of a protective effect for *Bacillus* strains when catfish were maintained in flow-through conditions after immersion challenge is likely a consequence of the removal of the *Bacillus* cells from the aquarium water, thereby preventing the persistence of the *Bacillus* cells within the aquarium that would naturally occur within an aquaculture pond. This suggests that a more pond-like environment wherein the probiotic is maintained within the water, and potentially the skin and gills of the fish, may be more conducive for effective biological control of disease. Since a static aquarium system is a better model for evaluating the biological control of disease that would occur in an aquaculture pond, we considered the results of the first disease challenge more relevant to the evaluation of *Bacillus* strains for their future adoption in pond-scale aquaculture.

Considering the importance of striped catfish for the Vietnamese aquaculture industry, the best performing *Bacillus* strains were sent to collaborators in Vietnam to evaluate their biological control activity for striped catfish against *E. ictaluri*. Significant reductions in mortality were obtained in this experiment, with an especially large reduction in mortality observed for fish fed with feed amended with *Bacillus* strain AP79. The biological control activity observed for *Bacillus* strains in this striped catfish disease challenge was comparable with previous studies in other fish species [17], [20]. However, in this study fish were fed with *Bacillus*-amended feed for two weeks before challenge, which is a relatively short period of time compared with the feeding duration time of one to two months in previous studies [16], [20]. The duration of time for probiotic feeding is a factor that can influence the degree of protection activity against infection in challenge [17]. Thus, the degree of protection afforded by the *Bacillus* strains in this study might be even better if a longer feeding duration was adopted.

It is interesting that the *Bacillus* strains that showed a significant protective effect in the disease challenges were different for the two catfish species. This could reflect a biologically meaningful difference in the interactions between *Bacillus* strains and their respective host. Also, there could be unique tripartite interactions between host, pathogen, and probiotic bacteria that could be influenced by environmental factors. Clearly more research is needed to understand the complex interplay between host, pathogen and probiotic *Bacillus* strains, and how to manipulate the environment to achieve the optimal biological control of disease. Further studies using an aquarium disease model with static conditions need to be conducted to optimize important parameters for challenge such as dosage and timing with the best performing *Bacillus* strains, with subsequent studies at a pond-scale to evaluate biological control efficacy within an aquaculture pond ecosystem.

One of the safety requirements for live bacteria directly consumed by humans is the absence of any acquired resistance to clinically important antibiotics [37]. Although the *Bacillus* strains used in this research were not for direct consumption by humans, they might be consumed inadvertently, as their hosts were cultured for food. Thus, it is important to analyze antibiotic resistance in probiotic strains and to distinguish the natural resistance, which is one of the phenotypic characteristics of a species, and acquired (i.e., transferable) resistance, which is associated with occurrence of plasmids. Also, pathogenicity and enterotoxin production are closely associated with plasmids [38]. Each of the four selected strains was susceptible to a broad spectrum of antibiotics tested with very similar susceptibility profile. Strains AP79, AP193 and AB01 did not contain any plasmids, ensuring their inability to conjugally transfer any plasmid that might confer antibiotic resistance or other traits. Although a plasmid was extracted from AP143, draft genome sequences from this strain were used to identify plasmid sequences and there were no predicted genes involved in antibiotic resistance or virulence.

Diffusible antimicrobial compounds were clearly involved in the *in vitro* antagonistic activity observed in soft agar overlay and in diffusion tests. The relative importance of secondary metabolites for *in vivo* biological control is unknown compared to other mechanisms such as enhancement of immune response, competitive exclusion of pathogens and modulation of intestinal microbiota. Future studies will investigate the relative contribution of antibiotic compound(s) production to the biological control activity of the *Bacillus* strains by comparing the mortality reduction activity of transposon mutagenized *Bacillus* strains with no antagonism activity with the wild type strains in aquarium challenge experiments.

In conclusion, a collection of *Bacillus* strains was identified that are antagonistic to the primary pathogens of catfish and are beneficial to both channel catfish and striped catfish when administered on feed for the control of ESC and BNP, respectively. These bacteria have potential application in aquaculture as a cost-effective alternative to the current use of antimicrobial compounds.
